# Supplementary material for: A Likelihood Approach for Real-Time Calibration of Stochastic Compartmental Epidemic Models
Source: PLoS Comput Biol. 2017 Jan 17;13(1):e1005257. doi: 10.1371/journal.pcbi.1005257 (PMC5240920; doi:10.1371/journal.pcbi.1005257)
Supplement: S1 File — (TAR.GZ) [file pcbi.1005257.s014.tar.gz › HSPH_Online-SI-Revision/Additions/MSS11/n100-extreme-SEITR_noisy/n100-extreme-SEITR_noisy_NEW_table-4.pdf]

1	R0	{1.38654, 9.85565, 0.1}	{2.56757, 17.005, 0.}	{{2.56757, 17.005, 0.}, {2.56757, 17.005, 0.}}	{85.1785, 72.5403, 100.}	inf	7628.	11 029.1	{10 852.2, 11 190.5}	44.5877	0.160104	newly	7087.2	5578.26	{5456., 5688.}	21.291	0.104026	Reff	1.09645	2.0674	{2.06293, 2.07124}	88.5543	ar	92 379.	90 114.2	{89 929., 90 334.}	2.45165
2	R0	{2.36629, 12.2029, 0.1}	{2.39633, 8.9094, 0.}	{{1.737, 5.43578, 0.}, {2.74056, 10.6768, 0.}}	{12.9217, 27.8151, 100.}	inf	2781.	2024.7	{712.9, 2841.36}	28.2409	0.174917	newly	3034.	2679.01	{1731., 3260.}	15.6945	0.0832746	Reff	2.22479	2.31019	{1.70668, 2.62563}	12.3276	ar	99 022.	85 749.1	{70 839., 92 090.}	13.404
3	R0	{2.32253, 12.836, 1.}	{2.15592, 5.62725, 0.}	{{1.65456, 3.37285, 0.}, {2.77394, 8.24535, 0.}}	{20.0013, 56.1604, 100.}	inf	2637.	1219.65	{802.27, 1846.47}	53.7485	0.360097	newly	2684.	3168.94	{2504., 3773.}	21.7683	0.0837491	Reff	2.19238	2.10454	{1.61811, 2.69767}	19.1687	ar	98 997.	80 448.2	{67 198., 92 306.}	18.7367
4	R0	{1.79075, 11.2058, 1.}	{2.58908, 9.38332, 0.02}	{{2.59708, 9.39342, 0.}, {2.59708, 9.39342, 0.}}	{44.581, 16.4364, 98.}	inf	4060.	3683.56	{3555., 3867.7}	10.2599	0.0488765	newly	3933.	4728.16	{4511., 5012.}	20.569	0.0808388	Reff	1.61667	2.43005	{2.43158, 2.44207}	50.312	ar	96 920.	90 232.7	{90 231., 90 724.}	6.89977
5	R0	{2.26952, 9.62371, 0.1}	{2.19039, 5.81963, 0.}	{{1.65456, 3.37285, 0.}, {2.77394, 8.24535, 0.}}	{18.1557, 39.5283, 100.}	inf	1974.	1202.39	{736.366, 1879.41}	39.2145	0.24449	newly	3231.	2990.95	{2093., 3649.}	14.5221	0.0720515	Reff	2.17415	2.13987	{1.62416, 2.69765}	17.4034	ar	98 881.	81 613.3	{67 766., 92 365.}	17.4631
6	R0	{1.57191, 6.89004, 0.1}	{2.80969, 9.61802, 0.}	{{2.14326, 6.48739, 0.}, {2.99092, 10.3908, 0.}}	{78.744, 41.6969, 100.}	inf	1965.	2498.82	{1561.88, 2988.48}	35.0902	0.132786	newly	3690.	3461.35	{2951., 3907.}	9.16233	0.042508	Reff	1.49482	2.69798	{2.08111, 2.85934}	80.4884	ar	95 046.	91 863.9	{83 338., 94 034.}	3.34801
7	R0	{1.99226, 17.2339, 1.}	{2.02235, 10.6804, 0.}	{{2.02235, 10.6804, 0.}, {2.02235, 10.6804, 0.}}	{1.51058, 38.0268, 100.}	inf	13 320.	7967.89	{7773.06, 8145.76}	40.181	0.223225	newly	6848.	6401.57	{6251., 6567.}	6.51913	0.0293621	Reff	1.39466	1.6696	{1.66579, 1.67278}	19.7137	ar	97 992.	80 286.1	{79 955., 80 585.}	18.0687
8	R0	{2.63369, 15.1879, 0.1}	{2.48777, 8.8973, 0.}	{{1.98388, 6.25659, 0.}, {2.80387, 10.6536, 0.}}	{9.4175, 41.4185, 100.}	inf	5972.	3247.76	{1976.62, 3883.}	45.6168	0.279554	newly	4729.	4364.41	{3726., 4946.}	10.21	0.0479427	Reff	2.31741	2.34797	{1.90114, 2.64249}	11.3464	ar	99 451.	87 961.6	{79 224., 92 537.}	11.5528
9	R0	{2.53271, 9.90552, 0.1}	{2.56202, 8.84524, 0.02}	{{1.9862, 6.16453, 0.}, {2.99092, 10.3908, 0.}}	{17.276, 18.4297, 116.}	inf	1262.	579.226	{180.087, 823.782}	54.1025	0.423905	newly	2227.	923.27	{386., 1250.}	58.542	0.4672	Reff	2.46668	2.53692	{1.97654, 2.95377}	17.6682	ar	99 355.	87 746.5	{79 262., 93 941.}	11.6839
10	R0	{2.48791, 18.9003, 2.}	{1.92851, 7.13705, 0.03}	{{1.74092, 5.83416, 0.}, {2.64721, 11.9965, 0.}}	{25.0891, 62.2384, 98.5}	inf	8409.	3069.84	{2396.37, 4940.95}	63.4934	0.455672	newly	4646.	4472.44	{4151., 4754.}	4.92036	0.0221069	Reff	2.05964	1.7966	{1.62714, 2.42995}	20.0056	ar	99 291.	75 168.4	{70 545., 91 090.}	24.2948
11	R0	{2.84414, 18.4765, 1.}	{2.64259, 13.5739, 0.}	{{2.15677, 10.1363, 0.}, {2.96947, 15.963, 0.}}	{11.0526, 26.5342, 100.}	inf	6430.	4194.21	{2739.44, 5054.95}	34.7712	0.195458	newly	4035.	3151.51	{2734., 3346.}	21.8957	0.108756	Reff	2.47881	2.45799	{2.04685, 2.74438}	10.3991	ar	99 654.	90 185.9	{83 571., 93 913.}	9.50094
12	R0	{2.29369, 14.622, 0.1}	{2.07322, 7.91511, 0.}	{{1.74092, 5.83416, 0.}, {2.13505, 8.31782, 0.}}	{9.61212, 45.8685, 100.}	inf	7900.	4244.63	{2564.53, 4646.32}	46.2705	0.27911	newly	5911.	5502.76	{4712., 5785.}	6.94942	0.0323851	Reff	1.91186	1.89466	{1.63324, 1.94897}	3.96682	ar	98 982.	80 869.4	{71 186., 83 327.}	18.2989
13	R0	{2.73413, 16.7529, 1.}	{2.58091, 10.4145, 0.}	{{1.53501, 4.02511, 0.}, {2.9884, 12.8326, 0.}}	{16.3915, 37.8349, 100.}	inf	6760.	4103.68	{1572.59, 5264.29}	39.2946	0.250273	newly	4789.	4358.4	{3867., 4681.}	9.01023	0.0422036	Reff	2.35852	2.39636	{1.46898, 2.75764}	17.7747	ar	99 543.	86 976.7	{61 314., 94 082.}	12.624
14	R0	{1.79554, 16.5943, 2.}	{2.11134, 12.6473, 0.}	{{2.11132, 12.644, 0.}, {2.11132, 12.644, 0.}}	{17.5881, 23.7852, 100.}	inf	10 085.	7353.41	{7192.58, 7490.}	27.0856	0.137294	newly	5421.	5018.02	{4878., 5141.}	7.43368	0.0336897	Reff	1.3756	1.79224	{1.78811, 1.79516}	30.2879	ar	96 821.	82 501.7	{82 134., 82 814.}	14.7895
15	R0	{1.91429, 13.7771, 1.}	{2.77476, 18.4356, 0.}	{{2.48143, 15.7026, 0.}, {2.93978, 19.9817, 0.}}	{44.95, 33.8288, 100.}	inf	8687.	9446.46	{7836.09, 10 393.4}	15.1978	0.0625869	newly	6031.	4578.85	{4484., 4676.}	24.0781	0.119723	Reff	1.53354	2.34076	{2.12756, 2.46354}	52.638	ar	97 541.	91 932.2	{88 851., 93 737.}	5.75024
16	R0	{1.42094, 10.5871, 2.}	{2.11519, 9.5888, 0.01}	{{1.24751, 2.5364, 0.}, {2.44101, 12.0941, 0.}}	{54.2289, 27.1803, 99.5}	inf	5617.	5540.07	{1249.56, 7734.06}	37.0571	0.216565	newly	4920.	5228.42	{4196., 6052.}	12.8622	0.0535578	Reff	1.20628	1.86564	{1.1562, 2.11694}	56.4748	ar	93 044.	75 670.6	{36 784., 88 726.}	18.8085
17	R0	{1.45515, 6.16466, 0.1}	{2.28105, 6.29497, 0.}	{{1.88643, 4.56746, 0.}, {2.77394, 8.24535, 0.}}	{56.7573, 22.9674, 100.}	inf	976.	1190.79	{731.333, 1823.43}	39.7752	0.142533	newly	2188.	2729.5	{1694., 3413.}	33.9232	0.127854	Reff	1.41733	2.23082	{1.84907, 2.69984}	57.3955	ar	93 254.	83 944.9	{76 177., 92 340.}	9.98248
18	R0	{2.85669, 13.2416, 0.1}	{2.46062, 8.76575, 0.01}	{{1.98388, 6.25659, 0.}, {2.91001, 10.6536, 0.}}	{14.4252, 33.8014, 108.}	inf	2472.	1066.37	{655.808, 1759.43}	56.8621	0.3964	newly	2807.	1534.41	{1099., 2204.}	45.4382	0.277436	Reff	2.71777	2.41494	{1.95474, 2.82472}	12.5725	ar	99 644.	87 129.	{79 063., 93 377.}	12.5597
19	R0	{2.56417, 17.9558, 0.1}	{2.42428, 13.0041, 0.}	{{2.14747, 10.7247, 0.}, {2.73108, 15.6124, 0.}}	{10.9675, 27.5771, 100.}	inf	6704.	3994.47	{3248.2, 4609.23}	40.4167	0.229904	newly	4054.	3034.11	{2824., 3297.}	25.1576	0.12679	Reff	2.21442	2.25317	{2.0089, 2.53293}	10.1795	ar	99 389.	87 492.3	{83 047., 91 955.}	11.9698
20	R0	{2.27517, 11.1864, 0.1}	{2.82174, 9.68713, 0.}	{{2.14326, 6.48739, 0.}, {2.99092, 10.3908, 0.}}	{25.8788, 13.4026, 100.}	inf	3074.	2508.05	{1562.82, 3030.73}	18.6501	0.0995206	newly	3692.	3444.52	{2950., 3937.}	9.91062	0.0465223	Reff	2.12451	2.70954	{2.08104, 2.85869}	28.2006	ar	98 894.	92 013.6	{83 214., 94 034.}	6.95735
21	R0	{2.35735, 10.4735, 0.1}	{2.22792, 6.20258, 0.02}	{{1.88643, 4.56746, 0.}, {2.77394, 8.24535, 0.}}	{15.9077, 40.8211, 116.}	inf	1612.	1040.46	{681.472, 1770.06}	39.9051	0.23904	newly	2417.	2494.83	{1491., 3403.}	30.4001	0.137204	Reff	2.2751	2.18418	{1.84911, 2.6985}	15.1016	ar	99 036.	82 932.	{75 996., 92 314.}	16.2607
22	R0	{1.79551, 11.9179, 2.}	{1.85868, 6.45564, 0.03}	{{1.47608, 3.83841, 0.}, {2.13505, 8.31782, 0.}}	{17.4014, 46.5456, 98.5}	inf	6986.	3542.3	{1933.19, 4650.86}	49.5115	0.328646	newly	5958.	5519.14	{4922., 6031.}	9.33803	0.0429792	Reff	1.5037	1.70311	{1.37653, 1.94938}	19.461	ar	97 033.	71 940.9	{56 283., 83 296.}	25.8593
23	R0	{1.23545, 6.31687, 0.1}	{2.48355, 9.2927, 0.}	{{2.26582, 8.12562, 0.}, {2.74056, 10.6768, 0.}}	{101.024, 47.1093, 100.}	inf	4010.	6200.59	{4990.15, 7637.36}	54.6283	0.181299	newly	6598.	7213.95	{6717., 7793.}	9.36329	0.038072	Reff	1.0892	2.21242	{2.04599, 2.41067}	103.124	ar	88 739.	88 552.4	{85 413., 92 045.}	3.53966
24	R0	{2.24089, 10.1789, 0.1}	{2.33231, 8.41798, 0.}	{{1.53501, 4.02511, 0.}, {2.80387, 10.6536, 0.}}	{18.2957, 22.0377, 100.}	inf	1397.	797.834	{247.379, 1203.91}	44.3753	0.329268	newly	2027.	1158.71	{541., 1585.}	43.5012	0.297011	Reff	2.17337	2.29844	{1.52358, 2.75277}	18.9095	ar	98 868.	83 406.9	{60 421., 92 540.}	15.6382
25	R0	{1.57298, 10.2607, 0.1}	{2.36349, 11.2216, 0.01}	{{2.32891, 10.9956, 0.}, {2.52588, 12.5037, 0.}}	{51.4969, 13.8814, 108.}	inf	3948.	4277.35	{3982.5, 5275.2}	13.2173	0.0635566	newly	3941.	3828.9	{3574., 4370.}	6.73941	0.0302112	Reff	1.41523	2.18134	{2.1521, 2.33251}	55.0633	ar	94 957.	86 245.5	{86 515., 89 703.}	9.17419
26	R0	{1.2154, 8.2576, 0.1}	{1.9505, 8.69558, 0.}	{{1.93419, 8.56054, 0.}, {1.93419, 8.56054, 0.}}	{60.4825, 5.30391, 100.}	inf	5890.	6767.38	{6534.71, 6844.09}	14.8961	0.0591299	newly	6408.	6956.81	{6804., 7132.}	8.571	0.035615	Reff	0.993966	1.65181	{1.63599, 1.64266}	66.1839	ar	88 421.	78 038.2	{77 332., 78 279.}	11.9114
27	R0	{2.22841, 18.7983, 2.}	{2.61123, 14.1646, 0.}	{{1.93419, 8.56054, 0.}, {2.7499, 15.3124, 0.}}	{21.668, 24.6496, 100.}	inf	13 535.	10 051.7	{6693.66, 10 871.3}	25.7359	0.135326	newly	6511.	6524.63	{6322., 6954.}	2.54201	0.0108797	Reff	1.58429	2.15246	{1.64069, 2.26216}	35.8627	ar	98 756.	89 625.6	{77 875., 92 210.}	9.2454
28	R0	{1.27778, 5.36767, 1.}	{2.0258, 5.03872, 0.01}	{{1.65456, 3.37285, 0.}, {2.77394, 8.24535, 0.}}	{58.5409, 27.8628, 99.}	inf	1253.	1153.3	{753.161, 1747.49}	26.0733	0.122126	newly	2971.	3320.73	{2687., 3877.}	16.0629	0.0647643	Reff	1.23118	1.97754	{1.61773, 2.69671}	60.6214	ar	89 843.	77 765.1	{66 753., 92 280.}	14.5167
29	R0	{2.38042, 15.8901, 2.}	{2.13534, 7.81601, 0.01}	{{1.737, 5.43578, 0.}, {2.9884, 12.8326, 0.}}	{23.3449, 50.8121, 99.5}	inf	5246.	2876.2	{1561.93, 5249.75}	45.4336	0.31993	newly	3642.	3886.97	{3117., 4647.}	14.4857	0.0599806	Reff	2.11469	2.00933	{1.65867, 2.75008}	20.9971	ar	99 112.	78 517.9	{70 281., 93 982.}	20.7786
30	R0	{2.53188, 12.7607, 0.1}	{2.51198, 8.11988, 0.01}	{{1.9862, 6.16453, 0.}, {2.99092, 10.3908, 0.}}	{16.2376, 36.3681, 108.}	inf	1411.	701.224	{395.538, 862.338}	50.7461	0.335168	newly	1649.	1317.41	{753., 2028.}	34.8969	0.198811	Reff	2.45506	2.48212	{1.97806, 2.95413}	16.5933	ar	99 317.	87 696.7	{79 592., 93 988.}	11.7002
31	R0	{1.24482, 10.5471, 1.}	{2.29055, 13.7413, 0.}	{{2.29055, 13.7413, 0.}, {2.29055, 13.7413, 0.}}	{84.0066, 30.2852, 100.}	inf	8518.	11 688.8	{11 507.6, 11 828.1}	37.2246	0.137406	newly	6713.	7082.1	{6953., 7194.}	5.49829	0.0231994	Reff	0.914831	1.7584	{1.75477, 1.76233}	92.2101	ar	89 097.	86 085.9	{85 820., 86 336.}	3.37959
32	R0	{2.30446, 17.9607, 1.}	{1.68323, 5.18765, 0.}	{{1.47608, 3.83841, 0.}, {1.74592, 5.58477, 0.}}	{26.9577, 71.1167, 100.}	inf	9835.	3165.62	{1907.57, 3753.96}	67.8127	0.509225	newly	5244.	6004.98	{4865., 6684.}	17.7662	0.0703646	Reff	1.82382	1.5422	{1.37806, 1.59289}	15.967	ar	99 028.	67 320.4	{56 333., 71 641.}	32.0188
33	R0	{2.31579, 11.8236, 2.}	{1.77413, 3.74971, 0.4}	{{1.39162, 1.89883, 0.}, {2.49133, 6.2492, 1.}}	{26.8769, 68.2862, 80.}	inf	4713.	1896.3	{976.434, 3778.08}	59.7644	0.456451	newly	5187.	6684.6	{4101., 8947.}	37.6707	0.136184	Reff	2.08296	1.68912	{1.32883, 2.33068}	24.3693	ar	99 012.	66 743.3	{50 209., 89 064.}	32.5907
34	R0	{1.6678, 10.6744, 1.}	{1.73899, 5.48872, 0.}	{{1.737, 5.43578, 0.}, {1.74592, 5.58477, 0.}}	{4.42788, 48.5805, 100.}	inf	7126.	3780.13	{3469.49, 4077.08}	46.9529	0.276224	newly	6918.	6835.07	{6196., 7456.}	5.88479	0.0259401	Reff	1.37288	1.5728	{1.55951, 1.59238}	14.5619	ar	95 809.	70 848.4	{70 257., 71 556.}	26.0524
35	R0	{1.65476, 7.07559, 0.1}	{2.24412, 6.09762, 0.}	{{1.88643, 4.56746, 0.}, {2.77394, 8.24535, 0.}}	{35.6174, 25.725, 100.}	inf	1256.	1196.07	{751.703, 1831.27}	35.4525	0.164958	newly	2514.	2837.39	{1845., 3559.}	24.5032	0.101286	Reff	1.60409	2.19338	{1.84855, 2.69679}	36.7365	ar	95 746.	82 922.	{75 989., 92 306.}	13.3938
36	R0	{2.71807, 18.5959, 0.1}	{2.1968, 9.50915, 0.01}	{{1.44762, 4.00571, 0.}, {2.53075, 12.2207, 0.}}	{19.1779, 48.8643, 108.}	inf	5300.	2427.19	{1003.87, 3611.86}	54.2039	0.389065	newly	3163.	2687.57	{2069., 3321.}	17.4015	0.0889089	Reff	2.4286	2.09201	{1.39666, 2.42393}	13.8767	ar	99 540.	79 095.3	{54 580., 89 841.}	20.5392
37	R0	{2.6202, 12.7552, 0.1}	{2.33767, 10.5404, 0.01}	{{1.44762, 4.00571, 0.}, {2.86001, 13.5542, 0.}}	{13.2918, 20.0007, 108.}	inf	1833.	1167.33	{529.37, 1946.14}	39.1831	0.264983	newly	2178.	1217.93	{752., 1704.}	44.0803	0.276452	Reff	2.51914	2.28772	{1.42391, 2.76568}	12.0334	ar	99 426.	82 569.1	{54 798., 92 900.}	16.9542
38	R0	{1.47672, 9.81651, 1.}	{2.7211, 14.1446, 0.}	{{2.44101, 12.0941, 0.}, {2.96947, 15.963, 0.}}	{84.2662, 44.0899, 100.}	inf	6452.	8937.94	{7406.78, 10 329.1}	38.5298	0.136487	newly	6266.	6023.45	{5833., 6189.}	3.88015	0.0172965	Reff	1.23	2.31368	{2.11058, 2.49512}	88.1031	ar	93 815.	91 336.5	{88 319., 93 957.}	2.72205
39	R0	{1.90004, 12.3313, 1.}	{1.88641, 6.84108, 0.02}	{{1.74092, 5.83416, 0.}, {2.64721, 11.9965, 0.}}	{13.7422, 44.5227, 98.}	inf	5575.	2963.47	{2399.91, 4968.88}	46.8436	0.29144	newly	4980.	4477.96	{4228., 4744.}	10.1751	0.0469725	Reff	1.65284	1.75871	{1.62769, 2.42831}	9.17023	ar	97 413.	73 982.1	{70 403., 90 943.}	24.0531
40	R0	{2.64218, 18.5237, 2.}	{2.42249, 8.96749, 0.}	{{2.26582, 8.12562, 0.}, {2.74056, 10.6768, 0.}}	{10.7723, 51.5891, 100.}	inf	10 912.	5905.52	{4973.57, 7587.54}	45.8805	0.27397	newly	6088.	7131.6	{6680., 7799.}	17.1419	0.0680028	Reff	2.0572	2.16642	{2.04513, 2.40693}	5.82659	ar	99 474.	87 712.1	{85 442., 91 973.}	11.8241
41	R0	{1.6945, 7.17996, 0.1}	{2.32274, 9.2679, 0.02}	{{1.514, 5.43578, 0.}, {2.93906, 12.8326, 0.}}	{42.1304, 44.0787, 116.}	inf	652.	336.896	{12.7061, 734.608}	53.4556	0.617205	newly	1333.	525.19	{10., 1022.}	63.1485	0.771534	Reff	1.66739	2.30831	{1.51384, 2.90838}	43.1487	ar	96 332.	80 935.4	{56 539., 93 582.}	15.9829
42	R0	{1.84559, 18.0074, 1.}	{2.02682, 10.771, 0.06}	{{2.02235, 10.6804, 0.}, {2.02235, 10.6804, 0.}}	{9.81971, 40.1858, 94.}	inf	13 082.	8000.77	{7805.89, 8174.32}	38.8414	0.213669	newly	6151.	6367.91	{6180., 6537.}	3.90587	0.0166011	Reff	1.29117	1.67256	{1.66581, 1.67471}	29.5382	ar	97 358.	80 413.9	{79 911., 80 807.}	17.4039
43	R0	{2.24965, 17.0361, 2.}	{2.1733, 8.77915, 0.}	{{1.74092, 5.83416, 0.}, {2.64721, 11.9965, 0.}}	{20.3841, 48.4674, 100.}	inf	7148.	3711.34	{2489.69, 5090.41}	48.0787	0.309378	newly	4281.	4454.72	{4269., 4691.}	5.13198	0.0217332	Reff	1.90728	2.01283	{1.62856, 2.43318}	20.8196	ar	98 770.	80 426.2	{70 566., 91 154.}	18.5723
44	R0	{2.54324, 15.7967, 0.1}	{2.25327, 9.32028, 0.}	{{1.74092, 5.83416, 0.}, {2.64721, 11.9965, 0.}}	{15.9803, 40.9985, 100.}	inf	7681.	3920.17	{2490.53, 5061.76}	48.9628	0.314194	newly	5449.	4442.45	{4254., 4640.}	18.4722	0.0889783	Reff	2.13836	2.08327	{1.62938, 2.43316}	17.8658	ar	99 354.	82 381.2	{70 750., 91 185.}	17.0832
45	R0	{2.54943, 14.8032, 0.1}	{2.43665, 8.62902, 0.01}	{{1.98388, 6.25659, 0.}, {2.80387, 10.6536, 0.}}	{10.1844, 41.7084, 108.}	inf	5633.	3157.85	{1918.22, 3873.58}	43.9401	0.268615	newly	4552.	4349.47	{3622., 4950.}	10.234	0.0468812	Reff	2.25387	2.30104	{1.90037, 2.64163}	12.3712	ar	99 380.	87 176.6	{79 019., 92 557.}	12.2795
46	R0	{2.37755, 12.6461, 2.}	{2.29001, 6.19583, 0.}	{{1.65456, 3.37285, 0.}, {2.77394, 8.24535, 0.}}	{19.3538, 51.006, 100.}	inf	2586.	1332.89	{799.447, 1861.3}	48.4573	0.314503	newly	2717.	3146.47	{2617., 3592.}	18.6305	0.0737382	Reff	2.25026	2.23323	{1.62356, 2.69893}	19.3737	ar	99 096.	83 155.4	{67 328., 92 394.}	16.086
47	R0	{1.69279, 11.6636, 1.}	{2.52588, 12.2207, 0.}	{{2.52588, 12.2207, 0.}, {2.52588, 12.2207, 0.}}	{49.2143, 4.77662, 100.}	inf	5607.	5335.19	{5168.39, 5482.07}	4.86109	0.0217465	newly	4864.	4427.02	{4269., 4569.}	8.98396	0.0410266	Reff	1.4668	2.29561	{2.29112, 2.29976}	56.5042	ar	96 214.	89 605.6	{89 397., 89 847.}	6.86844
48	R0	{1.82402, 10.0038, 1.}	{2.55119, 9.58345, 0.}	{{2.26582, 8.12562, 0.}, {2.74056, 10.6768, 0.}}	{39.8661, 11.9003, 100.}	inf	2604.	2558.11	{1878.42, 3092.92}	15.5813	0.0710749	newly	3183.	3226.52	{2838., 3748.}	9.16934	0.0387961	Reff	1.71321	2.44104	{2.18304, 2.62462}	42.4834	ar	97 076.	89 234.9	{85 342., 92 097.}	8.07723
49	R0	{1.6424, 11.1667, 2.}	{1.56554, 4.21652, 0.}	{{1.53501, 4.02511, 0.}, {1.53501, 4.02511, 0.}}	{7.87455, 62.2402, 100.}	inf	8869.	3339.91	{2977.58, 3372.83}	62.3418	0.434481	newly	7611.	7494.05	{6992., 7879.}	4.36776	0.0190907	Reff	1.28365	1.40381	{1.37621, 1.38572}	9.36105	ar	95 546.	61 511.3	{59 583., 61 312.}	35.6213
50	R0	{2.05638, 14.3048, 1.}	{2.3835, 9.25936, 0.33}	{{2.26582, 8.12562, 0.}, {2.75598, 12.4651, 1.}}	{16.7909, 35.271, 67.}	inf	6927.	5321.35	{4880.26, 6118.47}	23.7646	0.121798	newly	5024.	6318.18	{5065., 7044.}	26.4797	0.098883	Reff	1.73898	2.15427	{2.04212, 2.48777}	24.2155	ar	98 194.	86 887.9	{85 258., 92 147.}	11.5141
